# Infrared spectroscopy of the nitrogenase MoFe protein under electrochemical control: potential-triggered CO binding†
†Electronic supplementary information (ESI) available: Further details of experimental methods, diagram and description of the electrochemical cell, proton reduction assay data, electrochemical data from control experiments on the Eu–L complexes alone, and a comparison of the current arising from wild type MoFe protein under N_2_ and Ar. See DOI: 10.1039/c6sc02860h


**DOI:** 10.1039/c6sc02860h

**Published:** 2016-10-27

**Authors:** P. Paengnakorn, P. A. Ash, S. Shaw, K. Danyal, T. Chen, D. R. Dean, L. C. Seefeldt, K. A. Vincent

**Affiliations:** a Department of Chemistry , University of Oxford , Inorganic Chemistry Laboratory , South Parks Road , Oxford , OX1 3QR , UK . Email: kylie.vincent@chem.ox.ac.uk; b Department of Chemistry and Biochemistry , Utah State University , Logan , Utah 84322 , USA; c Department of Biochemistry , Fralin Center , Virginia Tech University , Blacksburg , Virginia 24061 , USA

## Abstract

We demonstrate electrochemical control of the nitrogenase MoFe protein, in the absence of Fe protein or ATP, using europium(iii/ii) polyaminocarboxylate complexes as electron transfer mediators. This allows the potential dependence of proton reduction and inhibitor (CO) binding to the active site FeMo-cofactor to be established. Reduction of protons to H_2_ is catalyzed by the wild type MoFe protein and β-98^Tyr→His^ and β-99^Phe→His^ variants of the MoFe protein at potentials more negative than –800 mV (*vs.* SHE), with greater electrocatalytic proton reduction rates observed for the variants compared to the wild type protein. Electrocatalytic proton reduction is strongly attenuated by carbon monoxide (CO), and the potential-dependence of CO binding to the FeMo-cofactor is determined by *in situ* infrared (IR) spectroelectrochemistry. The vibrational wavenumbers for CO coordinated to the FeMo-cofactor are consistent with earlier IR studies on the MoFe protein with Fe protein/ATP as reductant showing that electrochemically generated states of the protein are closely related to states generated with the native Fe protein as electron donor.

## Abstract 2

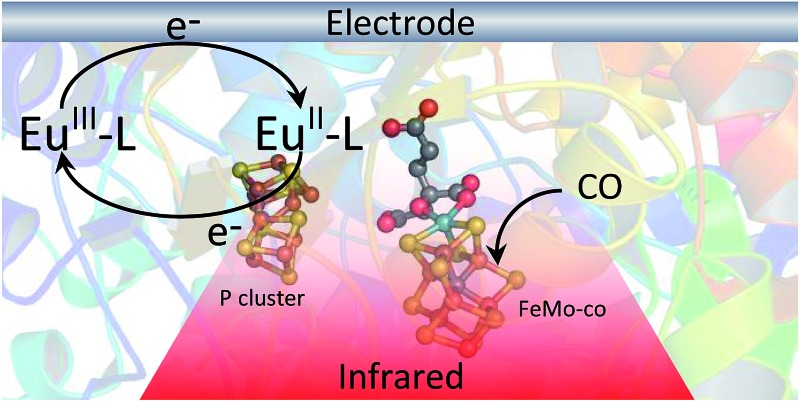
Electrochemical control over nitrogenase allows us to examine electrocatalytic proton reduction and potential-triggered CO inhibition using infrared spectroscopy.

## Introduction

**Scheme 1:**
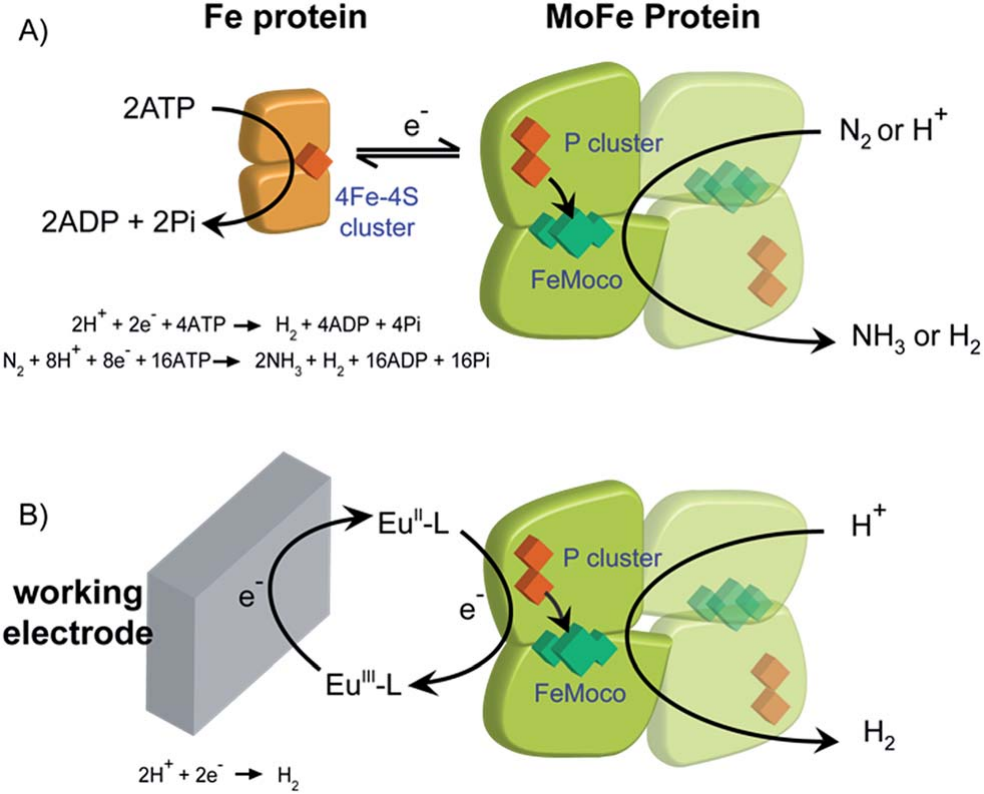
Electron delivery to nitrogenase MoFe protein. (A) ATP-coupled delivery of electrons *via* the Fe protein; (B) electron transfer to support proton reduction by the nitrogenase MoFe protein mediated by europium(iii/ii) polyaminocarboxylate complexes. L = polyaminocarboxylate ligand.

Biological dinitrogen fixation occurs in diazotrophic bacteria that contain the enzyme nitrogenase. In the Mo-dependent nitrogenase, two protein components work together to achieve the reduction of dinitrogen (N_2_) to ammonia (NH_3_). The molybdenum–iron (MoFe) protein (α_2_β_2_) houses the active site FeMo-cofactor [7Fe–9S–1Mo–C–homocitrate], FeMo-co, where substrates are reduced, and a [8Fe–7S] P cluster, that appears to function as an electron carrier. The Fe protein component (α_2_) contains a single [4Fe–4S] cluster and functions to deliver one electron to the MoFe protein during a transient association of the two proteins, in a process that is coupled to the hydrolysis of two equivalents of ATP for each electron transferred.^1^ The requirement for ATP hydrolysis by this system is energy intensive, comparable to the energy input required in the industrial Haber–Bosch process for N_2_ reduction where the energy comes primarily from fossil fuels. The complexity of the fully functioning nitrogenase system makes studies to define the mechanism of electron transfer and coupling with ATP challenging. The redox level of the MoFe protein is typically controlled indirectly by the ratio of the two component proteins (Scheme 1A), which complicates access to well defined, catalytically relevant, redox states of the MoFe protein.^2,3^ As a step toward controlled delivery of electrons to nitrogenase, and as a way to establish potential dependence of catalytically relevant events, it is desirable to gain electrochemical control of the MoFe protein without the Fe protein. In this direction, earlier reports have demonstrated Fe protein-independent electron transfer to variants of the nitrogenase MoFe protein with amino acid substitutions. Introduction of a cysteine-bound Ru photosensitizer at position 158 of the α chain was shown to allow light-driven reduction of protons, acetylene and hydrogen cyanide at very low rates.^4,5^ Recently, it was shown that CdS nanorods could be coupled to MoFe protein to enable photocatalytic N_2_ reduction to ammonia.^6^ Variants of the MoFe protein with amino acid substitutions in the region between the P cluster and FeMo-co have been shown to reduce protons to H_2_, azide to ammonia, and hydrazine to ammonia with low potential Eu^II^ polyaminocarboxylate complexes as reductants.^7^ A crystal structure of one of these variants bearing a Tyr to His substitution in the β chain of the MoFe protein (β-98^Tyr→His^) showed only minor structural changes that affected solvent ordering between the P cluster and FeMo-co, but no global structural changes.^8^ Here we show that it is possible to use the Eu^III/II^-ligand redox couples to mediate control by an electrode of wild type and amino acid substituted *Azotobacter vinelandii* nitrogenase MoFe protein, as evidenced by electrocatalytic proton reduction (Scheme 1B), and potential-dependent interaction of CO with the MoFe protein detected with infrared spectroelectrochemistry.

## Results and discussion

### Electrochemical and IR spectroelectrochemical cell for mediated electrochemistry of nitrogenase MoFe protein

**Fig. 1:**
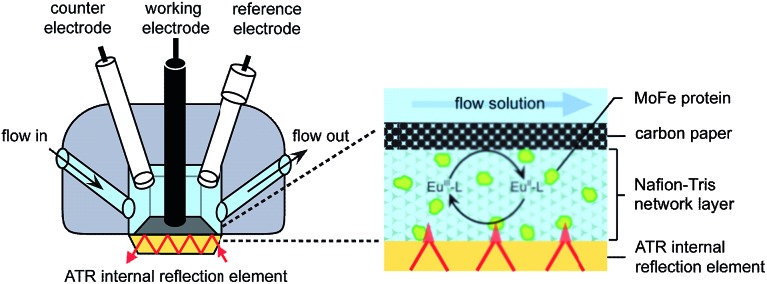
Schematic representation of the cell used for electrochemical and attenuated total reflectance (ATR)-IR spectroelectrochemical measurements on nitrogenase MoFe protein. A Pt wire counter electrode and a miniature saturated calomel reference electrode^14^ were inserted into buffered electrolyte solution in contact with a carbon paper working electrode pressed against a carbon rod connector. Protein was trapped in a Nafion–Tris layer between the working electrode and the ATR internal reflection element. Inset: cross section of the working electrode construction. Experimental details are provided in the ESI.†

Electrochemical experiments were conducted on samples of nitrogenase MoFe protein (*ca.* 2 nmol) contained within a layer of the polymer electrolyte, Nafion, titrated to pH 7.4 in Tris–HCl buffer (Nafion–Tris, see ESI Experimental methods†), in contact with a carbon paper working electrode, as shown in Fig. 1. Nafion allows movement of small molecules and ions within hydrated channels,^9^ and traps the protein within its structure. We have demonstrated previously that enzymes retain their native activity within an aqueous Nafion environment titrated to near-neutral pH.^10^ Assays for proton reduction activity and N_2_ reduction activity were conducted for both wild type and β-98^Tyr→His^ variant MoFe proteins in the presence and absence of Nafion as shown in Fig. S2 of the ESI.† These showed that more than 90% proton reduction activity and more than 80% N_2_ reduction activity is retained after exposure to Nafion. EPR spectra showed no change in signal intensity or linewidth for either the wild type or β-98^Tyr→His^ MoFe protein after exposure to Nafion (see discussion under ESI Fig. S2†).

For electrochemical and IR spectroelectrochemical experiments, europium(iii) together with three ligands was added into the Nafion–Tris and the electrolyte to give Eu–BAPTA, Eu–EGTA and Eu–DTPA each at 0.25 mM (collectively defined as Eu–L, see ESI for further details†). These complexes provide a convenient series of small-molecule electron transfer mediators with reduction potentials in water spanning the very negative potential range. Reduction potentials were measured at pH 8.0 as –634 mV (Eu–BAPTA), –868 mV (Eu–EGTA) and –1090 mV (Eu–DTPA) (see ESI, Fig. S3†) in agreement with literature values,^11,12^ and the Eu–DTPA potential is insensitive to pH above pH 4.0.^13^ We chose to utilise carbon as the working electrode because this material exhibits minimal background proton reduction current down to potentials as low as –1 V *vs.* the standard hydrogen electrode (SHE).^10^ The same cell design was used for both electrochemical and attenuated total reflectance (ATR)-IR spectroelectrochemical experiments (Fig. 1). Further detail is provided in the ESI Experimental methods.†

Flow of solution through the cell during experiments aided mass transport and allowed for exchange of gas saturated buffers. All experiments were conducted within an anaerobic glove box operating at <1 ppm O_2_.

### Electrocatalytic proton reduction by nitrogenase MoFe protein

**Fig. 2:**
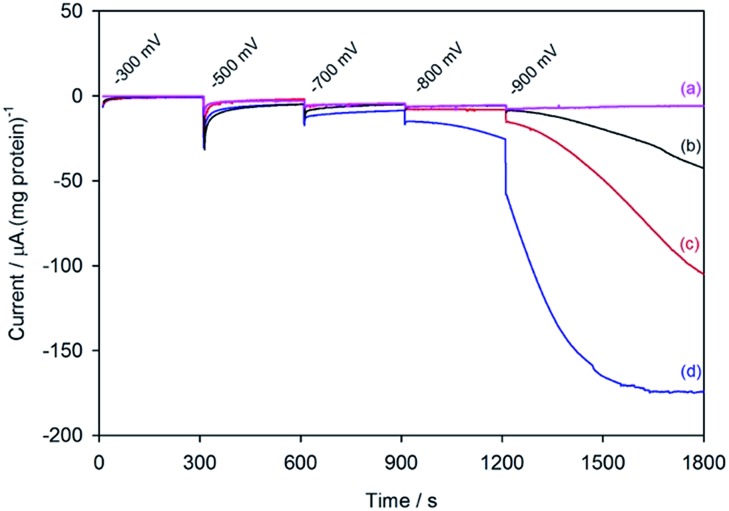
Current–time traces for electrochemical experiments on apo-MoFe protein (a); wild type MoFe protein (b); β-98^Tyr→His^ variant (c); and β-99^Phe→His^ variant (d), each in the presence of Eu–L mediator mixture. Potential steps were applied at the times indicated, and throughout the experiment, N_2_-flushed buffer (Tris–HCl, pH 7.4) flowed through the cell.

Fig. 2 shows the electrochemical response for different samples of the nitrogenase MoFe protein as the electrode is stepped to progressively more negative potentials under a N_2_ atmosphere. Potentials (*E*) are quoted relative to SHE *via* the conversion *E*_SCE_ = –0.242 V *vs. E*_SHE_ at 25 °C. To enable comparison between protein samples of slightly different concentrations, the currents are presented in units of μA (mg protein)^–1^. For MoFe protein lacking the active site FeMo-co (apo-protein, Fig. 2a) the stepwise increase in current at each potential is very small, and is consistent with the current observed for reduction of the Eu–L complexes alone (see ESI, Fig. S4†). For the wild type MoFe protein (Fig. 2b) the current is very close to the background level in the first potential steps, but a clear negative current is observed after stepping to –900 mV. The steady increase in current magnitude over time presumably reflects the protein equilibrating with the Eu–L pool as it becomes slowly more reduced. An analogous experiment on the wild type MoFe protein conducted under an Ar atmosphere gave almost indistinguishable current from that under N_2_ (see ESI, Fig. S5†), showing that the current at –900 mV does not arise from electrocatalytic reduction of N_2_ itself. We therefore attribute the current observed at –900 mV to enzyme-catalysed proton reduction mediated by the reduced Eu–L complexes.

Fig. 2 also shows electrochemical data for two variants of the MoFe protein with substitution of amino acids that are located between the P-cluster and the FeMo-cofactor. The response for the β-98^Tyr→His^ variant (Fig. 2c) is similar to that of the wild type, but with higher electrocatalytic current at –900 mV. Substitution of the nearby 99^Phe^ residue by His gives a variant, β-99^Phe→His^, that also shows higher electrocatalytic current than the wild type enzyme at –900 mV, and shows evidence of some electrocatalytic activity at –800 mV (Fig. 2d).

**Fig. 3:**
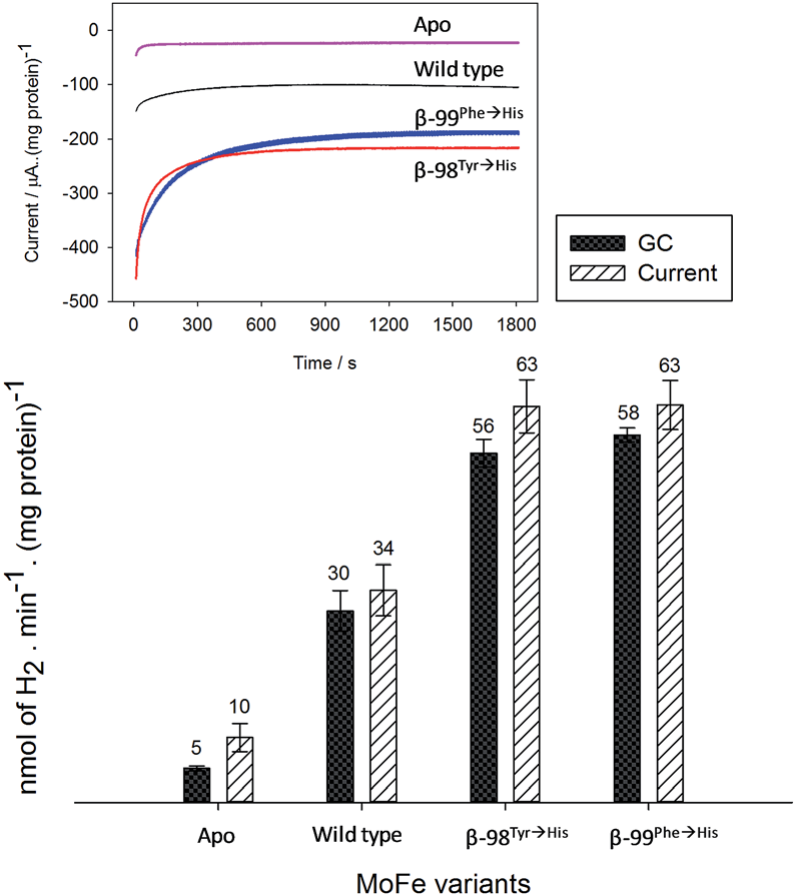
Confirmation of electrocatalytic proton reduction at –900 mV for electrodes modified with Nafion–Tris containing Eu–L and apo-MoFe protein; wild type; β-98^Tyr→His^ and β-99^Phe→His^ MoFe protein. Black columns show the headspace H_2_ detected by GC; hashed columns show the calculated amount of H_2_ obtained from integration of the charge passed during electrolysis over 30 minutes at –900 mV based on the assumption that 0.5 moles of H_2_ is produced per mole of electrons passed. Error bars represent the standard deviation for three repeat experiments. Inset: current–time traces for each electrode during the electrochemical poise.

Assignment of the electrochemical response at –900 mV as electrocatalytic proton reduction by the MoFe protein was confirmed in a separate experiment in a larger volume, stirred electrochemical cell (see ESI Experimental methods, Fig. S1†) in which H_2_ gas produced during a 30 minute poise at –900 mV was detected by gas chromatography (see ESI Experimental methods†). As shown in Fig. 3, there is good agreement between the calculated H_2_ produced on the basis of charge passed during the experiment (assuming 0.5 equivalents of H_2_ per electron) and the level of H_2_ detected in the headspace of the electrochemical cell, as measured by gas chromatography.

These results demonstrate electrochemically-controlled substrate reduction by nitrogenase. The rate of electrocatalytic H_2_ production is 30 ± 3 nmol min^–1^ (mg protein)^–1^ for wild type MoFe protein at –900 mV, representing approximately 1.4% of the rate observed in an assay with Fe protein and MgATP (2221 ± 66 nmol min^–1^ (mg protein)^–1^, see ESI Table S1†). The low electrocatalytic rate for the wild type protein is consistent with the observation that it has negligible activity in solution assays with Eu^II^–EGTA or Eu^II^–DTPA as electron donors.^7^ The fact that the low level of proton reduction by the apo-protein matches that of the control sample (‘no protein’) indicates that the FeMo-co centre within the wild type nitrogenase is responsible for its electrocatalytic proton reduction activity. Interestingly, the β-98^Tyr→His^ and β-99^Phe→His^ variants, which show significantly lower proton reduction activity than wild type nitrogenase in assays with the Fe protein as electron donor (see ESI, Table S1†), show higher electrocatalytic activity than the wild type, suggesting that the structural changes induced by these single-site substitutions equip the variants for more effective electron transfer with the reduced Eu–L mediators. The electrocatalytic rates observed for the β-98^Tyr→His^ and β-99^Phe→His^ variants represent 4–5% of the rates observed in proton reduction assays with Fe protein as electron donor (see ESI Table S1†). These results are in agreement with a previous report showing the ability of Eu(ii)–DTPA to support the two-electron reduction of hydrazine by the β-98^Tyr→His^ and β-99^Phe→His^ variants, but not wild type MoFe protein.^8^

At –300 mV, the most positive potential shown for the electrochemical experiments in Fig. 2, the FeMo-co centre should be in the so-called ‘resting state’, termed M^N^. Oxidation of this state to the ‘oxidised state’ termed M^OX^ has a midpoint potential of –42 mV. The midpoint potentials for reduction of FeMo-co below the resting state are not well understood. Assuming that the Eu–L mediator system does not introduce any significant overpotential to catalysis by the MoFe protein, the onset of electrocatalytic proton reduction in the region of –800 to –900 mV suggests an approximate reductive driving force required for catalysis by nitrogenase. This is consistent with the estimated potential likely to be experienced by the MoFe protein during photocatalysis on CdS nanorods,^6^ although it represents a significant overpotential relative to the H^+^/H_2_ couple potential (*E*°′ = –437 mV at pH 7.4).

### Effect of CO on proton reduction by the MoFe protein

We next investigated electrochemical control of binding of the well-established inhibitor carbon monoxide (CO) to the MoFe protein. Under assay conditions using Fe protein/ATP, CO is known to inhibit reduction of all substrates catalysed by nitrogenase,^3^ although inhibition of proton reduction by CO is weak and has only been detected at pH values above approximately pH 7.^15^ Proton reduction assays of wild type and the β-98^Tyr→His^ and β-99^Phe→His^ variants of the MoFe protein conducted at pH 7.4 with Fe protein as electron donor (ESI Table S1†) do not show significant inhibition by CO.

**Fig. 4:**
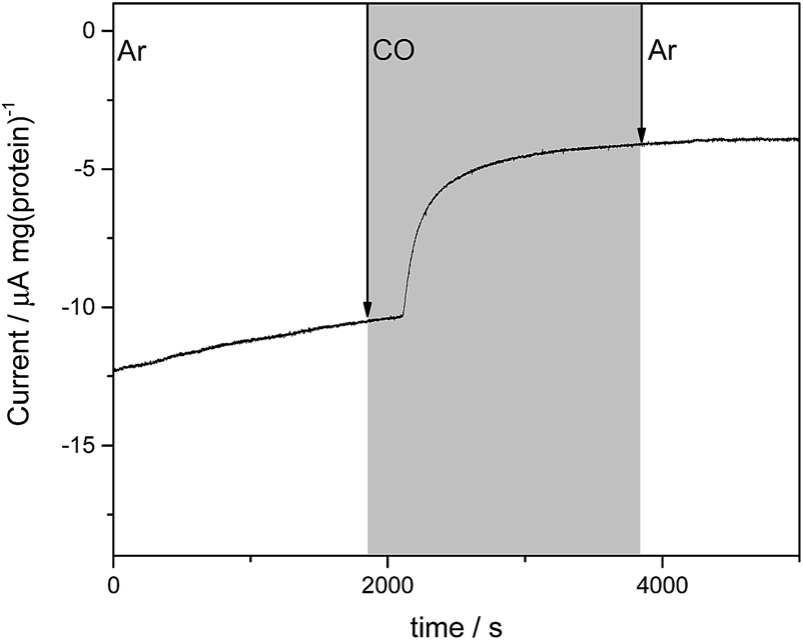
Current–time trace for an electrochemical experiment on β-98^Tyr→His^ MoFe protein showing the effect of CO on the electrocatalytic proton reduction current. During the period shown in grey, the cell was flushed with CO-saturated buffer solution (Tris–HCl, pH 7.4 containing Eu–L), while Ar-saturated buffer was flushed through the cell in the periods shown in white.

In electrochemical experiments on the MoFe protein with the Eu–L mediators at pH 7.4, CO is seen to inhibit proton reduction irreversibly at –900 mV, causing a substantial drop in the electrocatalytic current. A representative trace for the β-98^Tyr→His^ variant is shown in Fig. 4; similar responses are observed for the wild type and β-99^Phe→His^ proteins. In electrochemical experiments conducted under a CO atmosphere, no hydrocarbon product of CO reduction was detected from either the wild type MoFe or variant MoFe proteins, showing that the proteins are not electrocatalytically reducing CO under these conditions.

Although proton reduction activity in the MoFe protein is generally reported to be fairly insensitive to CO in assays, CO-bound states of the MoFe protein prepared during turnover studies with Fe protein and ATP under a CO atmosphere have been detected by both stopped-flow IR spectroscopy^16–18^ and freeze-quench EPR and ENDOR.^19^ Several CO-bound species were observed in these studies, depending on the CO concentration. A crystal structure of the MoFe protein prepared under turnover conditions under a CO atmosphere showed a single enzyme form, in which the FeMo-cofactor had opened up with a bridging CO between two Fe sites on a face of the cluster.^20^

**Fig. 5:**
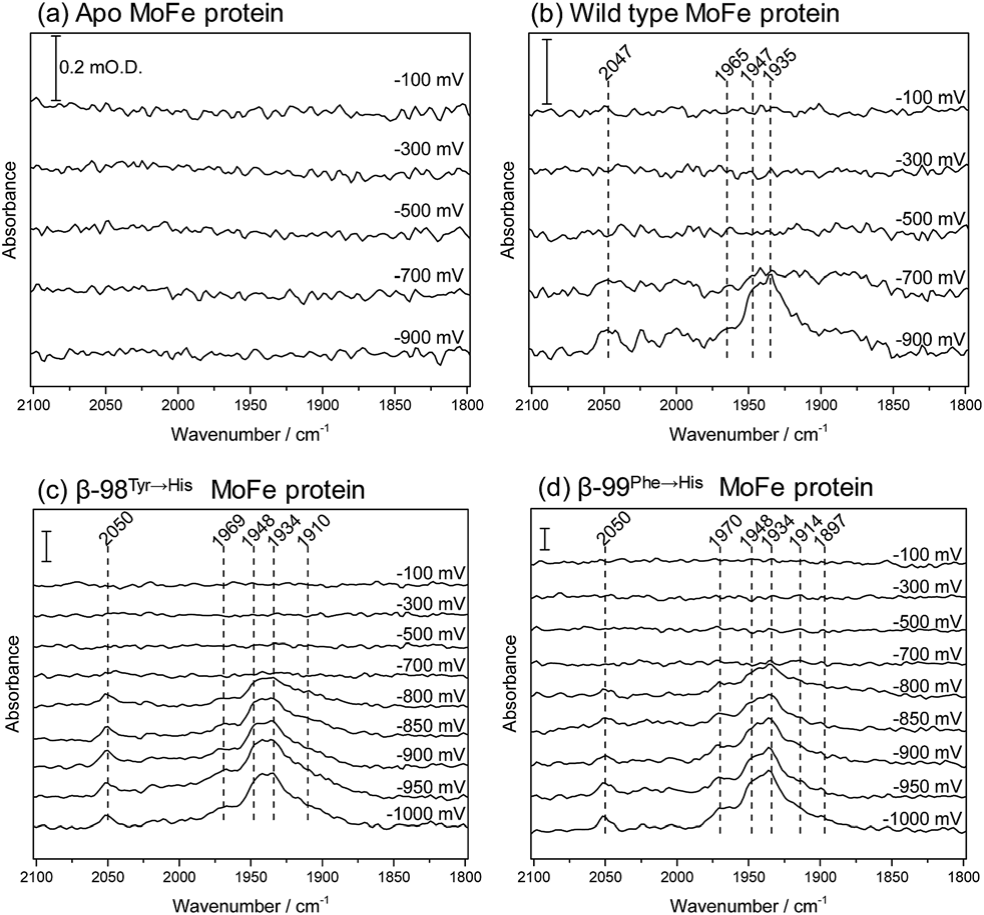
Infrared spectra recorded *in situ* in the ATR-IR spectroelectrochemical cell, showing the effect of CO on the MoFe protein as a function of potential: (a) apo protein; (b) wild type; (c) β-98^Tyr→His^; (d) β-99^Phe→His^ MoFe protein. Spectra were recorded following equilibration at the potentials indicated. Each spectrum is reported relative to a background spectrum recorded at –100 mV prior to addition of CO. Spectra represent 500 co-added interferograms (collected over 356 s), 4 cm^–1^ resolution. In each panel, the scale bar indicates 0.2 m O.D. absorbance.

We have applied ATR-IR spectroelectrochemistry to examine the effect of CO as a function of potential. Free CO in aqueous solution gives a weak IR stretching band (*ν*_CO_) at about 2143 cm^–1^, which typically shifts to lower wavenumber (*ca.* 2100–1800 cm^–1^) and becomes much more intense when CO coordinates to a metal centre, making it easy to determine features arising from CO ligands at metal sites.^21^ Potential-dependent difference spectra for the MoFe proteins, recorded under an atmosphere of 100% CO, are shown in Fig. 5, relative to a background spectrum recorded at –100 mV prior to introduction of CO to the cell. For the apo-protein (Fig. 5a), no *ν*_CO_ bands are observed at any of the applied potentials. The wild type MoFe protein and the β-98^Tyr→His^ and the β-99^Phe→His^ variants exhibit no *ν*_CO_ bands at the more positive potentials, but all show evidence of potential-induced coordination of CO at more negative potentials (Fig. 5, panels b–d). The general features of the CO-bound spectra are similar in all three cases: a broad band centred at approximately 1940 cm^–1^ dominates in intensity, with shoulders at both higher and lower wavenumber (approximately 1965–1970 and 1910–1914 cm^–1^, respectively), in addition to several features at higher wavenumber (>2000 cm^–1^). The absence of any detectable *ν*_CO_ bands for the apo protein provides strong evidence that all the *ν*_CO_ bands observed for the wild type and β-98^Tyr→His^ and β-99^Phe→His^ variants of the MoFe protein arise from CO bound at FeMo-co rather than the P-cluster.

The absence of *ν*_CO_ bands at potentials more positive than –700 mV shows that CO does not bind to resting state FeMo-co, but requires more reduced levels of the cofactor. This is consistent with studies on isolated FeMo-co in *N*-methyl formamide in which no perturbation of the oxidised/semi-reduced FeMo-co couple was observed under CO, and the onset of CO binding was only detected at potentials around –600 mV more negative than the oxidised/semi-reduced couple, coinciding with potentials at which electrocatalytic proton reduction by isolated FeMo-co is observed.^22,23^ In the spectra shown in Fig. 5, the onset for CO binding also coincides fairly closely with the onset potential for electrocatalytic proton reduction for each of the proteins (see Fig. 2), suggesting that states generated during the MoFe protein catalytic H^+^ reduction cycle may be targeted by CO. This is consistent with spectroscopic and crystallographic studies during turnover under CO, which all show CO binding to be dependent on catalysis.^16–20^ In this context, the weak inhibitory effect of CO observed in biochemical assays is curious and will be explored further in future work.

The spectra in Fig. 5 resemble quite closely the ‘hi-CO’ spectra reported in stopped flow IR studies on the MoFe protein reduced by Fe protein in the presence of ATP and CO, which are dominated by a band at 1936 cm^–1^, with smaller bands at 1906 and 1960 cm^–1^,^16,18^ as well as weak higher wavenumber features around 2020–2030 cm^–1^ which were not assigned.^18^ This provides compelling evidence that the electrochemically generated states of nitrogenase MoFe protein studied here are relevant to states generated using the native Fe protein as electron donor. In the spectra shown in Fig. 5, the band centred around 1940 cm^–1^ appears to have contributions from two components. These are most clearly resolved for the β-99^Phe→His^ protein, at 1948 and 1934 cm^–1^. A similar split *ν*_CO_ band and pattern of relative intensities was observed for isolated FeMo-co in the presence of imidazole which is presumed to mimic the native histidine coordination at molybdenum.^22,23^

Both the β-98^Tyr→His^ and β-99^Phe→His^ variants show more intense features than the wild type protein. This could be related to their improved ability to take up electrons from the Eu–L system, but could also arise from slight structural alterations that modify CO access to FeMo-co. Altered CO access has been observed in a variant of the MoFe protein with a mutated residue close to the terminal Fe site of FeMo-co.^24^

Previous spectroscopic studies of CO binding to FeMo-co within the MoFe protein have been interpreted in terms of an initial bridging CO ligand between two Fe sites that opens up to two terminal CO ligands at higher CO concentrations.^17,19,25–27^ Broad lower wavenumber features below 1900 cm^–1^ in the spectra in Fig. 5 may be associated with bridging CO ligands. Development of a peak at 1897 cm^–1^ in spectra of the β-99^Phe→His^ variant becomes clear in difference spectra following the onset of CO binding (see ESI, Fig. S6†).

Additionally, a higher wavenumber band at 2050 cm^–1^ is particularly sharp in the spectra of the β-99^Phe→His^ variant, and indicates CO bound to a less electron-rich site, possibly arising from CO on Mo. Molybdenum hexacarbonyl exhibits a single *ν*_CO_ at 2004 cm^–1^.^28^ For the relatively few characterised examples of carbonyl complexes of Mo in higher oxidation states, the *ν*_CO_ bands are very sensitive to the co-ligands. The Mo(iii) complex [CpMoCl(dppe)(CO)]^+^ has its *ν*_CO_ band at 2002 cm^–1^, while the Cp* derivative has *ν*_CO_ at 1971 cm^–1^.^29^ ‡
‡Cp = cyclopentadienyl; Cp* = pentamethyl cyclopentadienyl; dppe = diphenyl phosphinoethane. The reduced Mo(ii) forms of these complexes have *ν*_CO_ bands at 1853 cm^–1^ and 1835 cm^–1^ respectively.^29^ The Mo(iii) complexes ((Me_3_SiNCH_2_CH_2_)_3_N)Mo(CO) and ((C_6_F_5_NCH_2_CH_2_)_3_N)Mo(CO) exhibit lower wavenumber *ν*_CO_ bands, at 1859 cm^–1^ and 1889 cm^–1^ respectively, however.^30^

Flushing CO out of the cell, whilst poising the potential at –900 mV, does not lead to any noticeable decrease in intensity of the *ν*_CO_ bands, consistent with the fact that electrocatalytic H^+^ reduction current does not recover when CO is flushed out with Ar (see Fig. 4). Switching to a N_2_ atmosphere and stepping the potential to –100 mV after the experiment on wild type MoFe protein shown in Fig. 5b resulted in partial depletion of all *ν*_CO_ features (see ESI, Fig. S7†), showing that CO binding to the FeMo-co centre is somewhat reversible. The Eu–L mediator system used in this work will not effectively mediate the solution potential above approximately –400 mV (see ESI, Fig. S3†) which may explain the incomplete reversibility.

## Conclusions

The results presented here demonstrate that the nitrogenase MoFe protein can be placed under electrochemical control to drive substrate reduction or ligand binding using a Eu(iii/ii)–ligand mediated system, without the need for the Fe protein/ATP. The electrocatalytic rate of H_2_ evolution we measure is modest compared to the rate for H_2_ evolution with Fe protein/ATP as electron donor, or for photocatalytic H_2_ production by wild type MoFe protein on CdS (3000 nmol of H_2_ min^–1^ (mg protein)^–1^),^6^ but is greater than the rate observed in an assay with Eu–EGTA as electron donor without electrochemical regeneration (6.5 nmol of H_2_ min^–1^ (mg protein)^–1^).^8^

The Eu–L mediated electrocatalytic method was then used to examine the inhibitory effect of CO on electrocatalytic proton reduction by wild type and β-98^Tyr→His^ and β-99^Phe→His^ MoFe proteins using a combination of electrochemistry and infrared spectroelectrochemistry, showing the onset potential for H^+^ reduction and CO binding to be closely related. Several CO ligands appear to bind to the FeMo-co cluster, possibly at different redox levels, and give rise to IR *ν*_CO_ bands that closely resemble the potential-dependent features observed in earlier studies on isolated FeMo-co, as well as those observed for the MoFe protein under turnover conditions with Fe protein/ATP as reductant. This approach opens up opportunities for future work to examine, systematically, electron transfer and ligand binding reactions to nitrogenase MoFe protein without the complexities of the Fe protein/ATP as a reductant system.
