# Supplementary material for: Infrared spectroscopy of the nitrogenase MoFe protein under electrochemical control: potential-triggered CO binding
Source: Chem Sci. 2016 Oct 27;8(2):1500–5. doi: 10.1039/c6sc02860h (PMC5460594; doi:10.1039/c6sc02860h)
Supplement: Supplementary file 1 [file SC-008-C6SC02860H-s001.pdf]

Electronic Supplementary Material (ESI) for Chemical Science.
This journal is © The Royal Society of Chemistry 2016



# Electronic Supplementary Information

## Infrared spectroscopy of the nitrogenase MoFe protein under electrochemical control: potential-triggered CO binding

**P. Paengnakorn, P. A. Ash, S. Shaw, K. Danyal, T. Chen, D. R. Dean, L. C. Seefeldt, K. A. Vincent**

## Experimental Methods

### 1. Protein purification and activity determination

The wild type, β-98$^{Tyr\rightarrow His}$ and β-99$^{Phe\rightarrow His}$ variant MoFe proteins were expressed in *Azotobacter vinelandii* and purified by Q-sepharose anion-exchange chromatography with a linear NaCl gradient followed by a gel filtration chromatography protocol as described previously,[1-3] and concentrated to 80 – 100 mg mL$^{-1}$. Proteins were greater than 95% pure as judged by sodium dodecyl sulphate polyacrylamide gel electrophoresis with Coomassie blue staining.

The activity assay for proton reduction was performed using 0.1 mg MoFe protein in 100 mM MOPS buffer solution (pH 7.4) containing 6.7 mM $MgCl_2$, 30 mM phosphocreatine, 5 mM ATP, 0.2 mg/mL creatine phosphokinase and 1.3 mg/mL Bovine Serum Albumin (BSA) and 0.5 mg Fe protein. The reaction vial was incubated at 30 °C for 8 minutes.

**Table S1. Specific proton reduction activity by wild type, β-98$^{Tyr\rightarrow His}$ and β-99$^{Phe\rightarrow His}$ MoFe proteins, measured at pH 7.4 under an Ar or CO atmosphere (100%, 1 bar).**

| MoFe protein | Specific activity (nmol of $H_2$/min/mg MoFe protein) | |
|---|---|---|
| | Ar | CO |
| **Wild type** | 2221 ± 66 | 1986 ± 24 |
| **β-98$^{Tyr\rightarrow His}$** | 1138 ± 21 | 1323 ± 17 |
| **β-99$^{Phe\rightarrow His}$** | 1389 ± 27 | 1318 ± 13 |

### 2. Preparation of Eu(III/II)-ligand redox mediator cocktail, referred to as Eu-L

Three polyaminocarboxylate chelating ligands were used: 1,2-bis(*o*-aminophenoxy) ethane-*N,N,N',N'*-tetraacetic acid (BAPTA, Sigma-Aldrich), ethylene glycol-bis(2-aminoethylether)-*N,N,N′,N′*-tetraacetic acid (EGTA, Sigma-Aldrich) and diethylenetriamine- *N,N,N'N'',N''*-pentaacetic acid (DTPA, Alfa Aesar). All three ligands were dissolved in 0.25 M NaOH, at a concentration of 40 mM each. Aqueous $Eu^{3+}$ was obtained by dissolving $Eu_2O_3$ (Alfa Aesar) in

0.5 M HCl to give a $Eu^{3+}$ concentration of 40 mM. Equimolar mixtures of Eu(III)-BAPTA, Eu(III)-EGTA and Eu(III)-DTPA complexes were prepared by adding the ligand solution to the $Eu^{3+}$ solution in a 1:3 volume ratio. The Eu(III)-ligand redox mediator cocktail, Eu-L, was diluted to a total Eu(III) concentration of 0.25 mM before use.

### 3. Preparation of pH 7.4 Nafion-Tris suspension

Nafion® 117 perfluorinated ion exchange resin (Sigma Aldrich, aqueous suspension, 10% v/v) was mixed with an equal volume of 100 mM Tris-HCl buffer, pH 7.4 containing 100 mM NaCl. This mixture was then titrated to pH 7.4 using concentrated NaOH or HCl to give a Nafion-Tris suspension at pH 7.4 (with ca 5% v/v Nafion concentration).

### 4. Electrochemistry on nitrogenase samples for GC analysis of headspace gas

Electrochemical experiments on samples of nitrogenase MoFe protein for GC analysis of gaseous headspace products were conducted within a layer of the polymer electrolyte, Nafion-Tris, in contact with a carbon paper working electrode (ESI, Figure S1). First, the 5.0 µL dithionite-free protein sample (approximately 2 nmol of protein) was mixed with 2.5 µL Nafion-Tris suspension pH 7.4. Then, the mixture was deposited directly on a piece of carbon paper (AvCarb P50, 1.0x0.5 $cm^3$, Fuel Cell Earth) to form a Nafion-Tris-protein film on the working electrode and then allowed to partially dry. The carbon paper was attached to the working electrode pin *via* an o-ring seal, and the whole working electrode assembly was suspended in stirred, buffered electrolyte solution (100 mM Tris-HCl, pH 7.4; 100 mM NaCl) in a custom-made gas-tight electrochemical cell equipped with a Pt wire counter electrode in a vycor-tipped compartment and a saturated calomel reference electrode (SCE, BAS Inc). Potentials ($E$) are quoted relative to the standard hydrogen electrode (SHE) via the conversion ($E_{SCE}$ = -0.242 V *vs* $E_{SHE}$ at 25 °C). Eu-L solution was added into the electrolyte which was stirred throughout the experiment period. These electrochemical experiments were conducted within an Ar-filled glove box (Vacuum Atmospheres Company, <2 ppm $O_2$). Electrochemical control was provided by an Autolab 128N potentiostat.

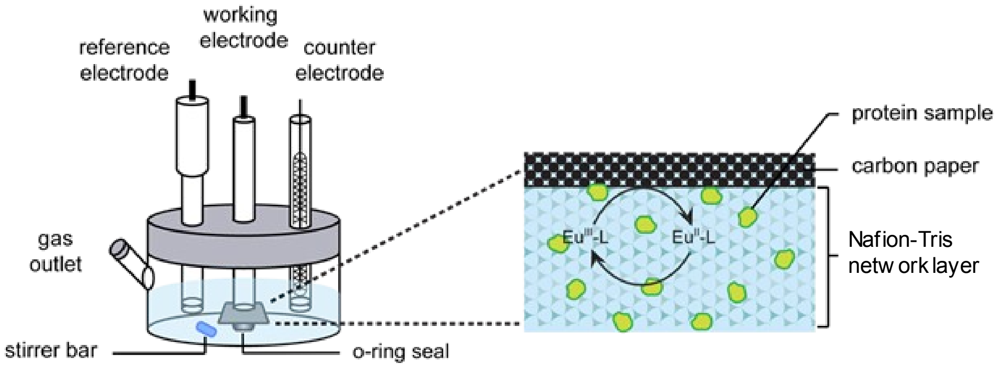


**Figure S1. A schematic diagram of electrochemical cell with three-electrode system (left). A cartoon showing protein trapped in Nafion network on carbon paper served as an extended working electrode (right).**

Product headspace gas was analysed by gas chromatography (GC-8A, SHIMADZU) using Ar carrier gas. $H_2$ gas was determined with a thermal conductivity detector (TCD) while hydrocarbon products were determined with a flame ionization detector (FID).

## 5. IR spectroelectrochemistry

The preparation of the spectroelectrochemistry (SEC) experiment is similar to that for electrochemical set up as described above. In the IR-SEC cell, a Nafion-Tris mixture containing approximately 2 nmol protein sample was directly deposited on an attenuated total reflectance (ATR) element (Si, 8.39x5x1 $mm^3$ with an incidence angle of 39°, Crystal GmbH), to give an initial protein concentration of approximately 0.25 mM. The film was allowed to partially dry, concentrating the sample to a final protein concentration of 0.75-1 mM. A piece of carbon paper placed in contact with the protein-Nafion film served as a working electrode. A Pt counter electrode and a mini SCE reference electrode were employed.[4] The cell body was filled with 0.25 mM Eu-L mediator solution in Tris-HCl buffer pH 7.4. For the spectroelectrochemical set up, all spectra were recorded inside an anaerobic glove box (Glove Box Technology) using a Varian 680-IR spectrometer equipped with a GladiATR™ accessory (PIKE technology, custom modified) and liquid $N_2$ cooled MCT detector. On the timeframe of the experiments presented in Figure 5, loss of protein from the Nafion film, as judged by loss of intensity for the amide I and II bands, was less than 1%.

**Supplementary Data**

**Effect of Nafion-Tris on nitrogenase activity for $H^+$ reduction and $N_2$ reduction in a solution assay with Fe protein and ATP.**

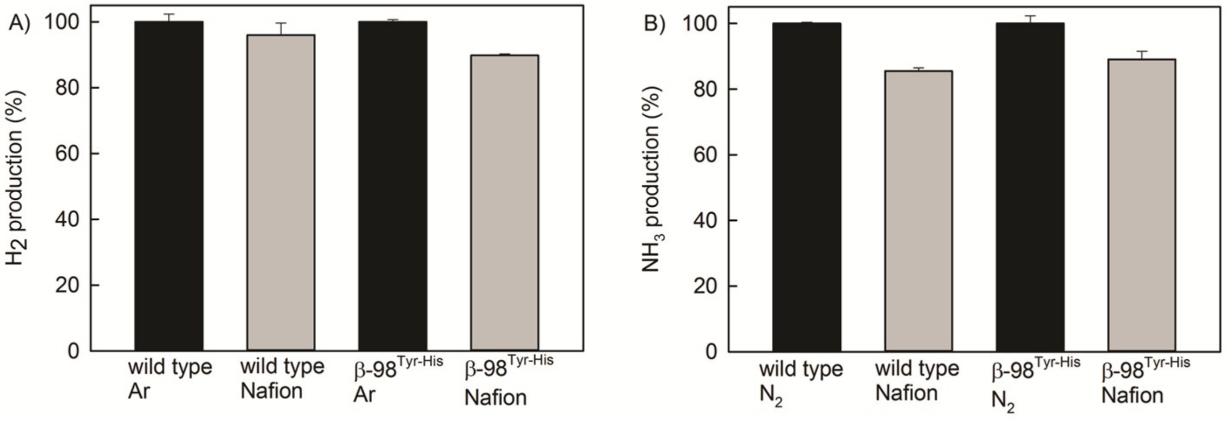


**Figure S2. Comparison of the relative activities for (A) proton reduction and (B) $N_2$ reduction of wild type and β-98$^{Tyr\rightarrow His}$ MoFe proteins in solution assays with (grey) and without (black) exposure to Nafion-Tris. For assays involving exposure to Nafion-Tris, 20 µL of MoFe protein was first incubated with 10 µL of Nafion-Tris for approximately 15 minutes to replicate conditions during electrochemical and IR spectroelectrochemical measurements. To determine the activity after this treatment, the mixture was then mixed with assay buffer to give a final MoFe protein concentration of 0.1 mg $mL^{-1}$ during the assays. The reaction was initiated by adding 0.5 mg of Fe protein to the assay buffer. The assay mixtures were incubated at 30 °C for 8 minutes and then quenched with 300 µL of 400 mM EDTA. Data are the average of three independent experiments; error bars represent ±2 standard deviations. Assays in panel A were conducted under an Ar atmosphere. Assays in panel B were conducted under a $N_2$ atmosphere.**

Substantial activity for both $H^+$ reduction and $N_2$ reduction is observed after incubation in Nafion-Tris. The small drop in relative activity for both reactions seen in the Nafion-Tris treated samples may simply be due to retarded diffusion of the Fe protein in the presence of Nafion-Tris. The greater drop in $N_2$ reduction activity compared to proton reduction activity in the Nafion-Tris treated samples likely reflects slowed diffusion of $N_2$ through the Nafion-Tris structure, relative to protons and $H_2$.

Further confirmation that the integrity of the FeMo-cofactor within the MoFe protein is retained following exposure to Nafion-Tris was provided by EPR measurements. To record EPR spectra on a sample exposed to Nafion-Tris, a 60 µL aliquot of MoFe protein (80 mg/mL) was added to 30 µL of Nafion-Tris and incubated for 15 min at room temperature. This mixture was then diluted to 400 µL with assay buffer to achieve a final MoFe protein concentration of 50 µM, and then transferred to a degassed X-band EPR tube. The sample was frozen in a liquid $N_2$/hexane slurry. EPR spectra were recorded as described previously.[5] The EPR spectrum for the sample exposed to Nafion-Tris showed no change in signal intensity or linewidth compared to a MoFe protein sample at the same concentration prepared without Nafion-Tris.

**Electrochemical characterisation of the Eu(III/II)-ligand complexes and $Eu^{3+}_{(aq)}$**

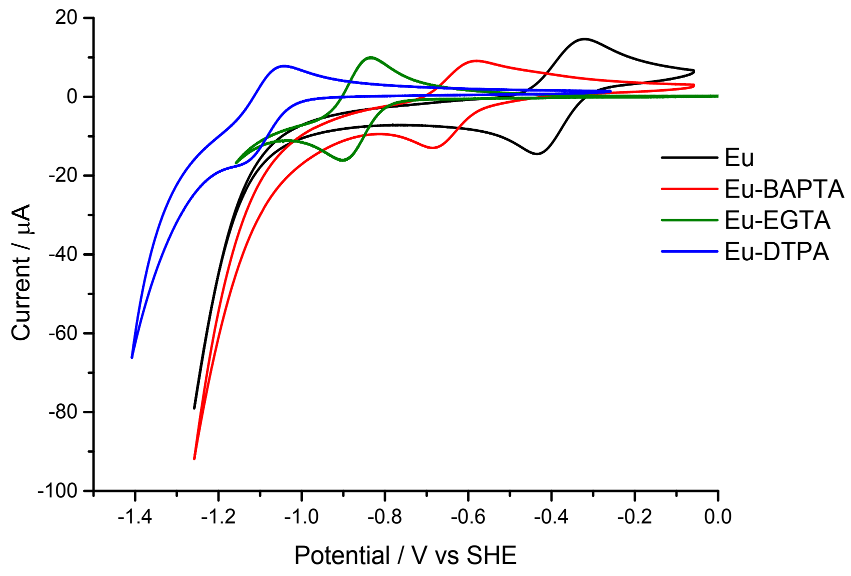


**Figure S3. Cyclic voltammograms of $Eu^{3+}_{(aq)}$ and $Eu^{III}$-ligand complexes (Eu-BAPTA, Eu-EGTA, and Eu-DTPA), each at 2.0 mM concentration. Voltammograms for $Eu^{3+}_{(aq)}$ (black), Eu-BAPTA (red) and Eu-EGTA (green) were performed in 50 mM Tris-HCl buffer pH 7.4, containing 50 mM NaCl as electrolyte. The voltammogram for Eu-DTPA (blue) was performed in 50 mM Tris-HCl buffer at pH 8.0. Measurements were performed in a glass electrochemical cell with a three-electrode set-up, comprising a glassy carbon working electrode, a platinum wire counter electrode and saturated calomel electrode (SCE) from BAS as the reference electrode. The scan rate was 20 mV $s^{-1}$. Electrochemical control was provided by an EcoChemie Autolab PGSTAT 128N potentiostat.**

**Proton reduction current for MoFe proteins compared to Eu-L and apo-MoFe protein control experiments**

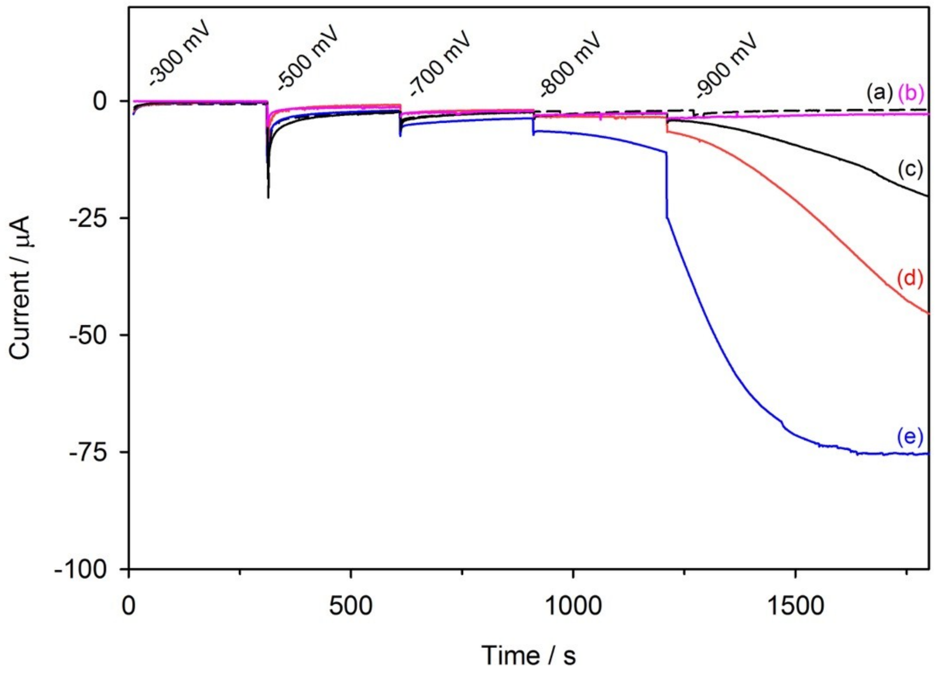


**Figure S4. Current-time traces of a blank Nafion-Tris-modified working electrode in Tris-HCl buffer pH 7.4 with 0.25 mM Eu-L mediator solution (a, dashed); compared to protein-containing Nafion-Tris-modified working electrodes: apo-MoFe protein (b, pink); wild type MoFe protein (c, solid black); β-99$^{His}$ variant (d, red) and β-98$^{His}$ variant (e, blue), each in the presence of 0.25 mM Eu-L mediator mixture. Potential steps were applied at the times indicated. Traces (b) to (e) show the same data presented in Figure 2 of the main text, but in units of current rather than current per mg of protein.**

**Proton reduction current for wild type MoFe protein under Ar and $N_2$ compared to the background current for Eu-L in the absence of protein.**

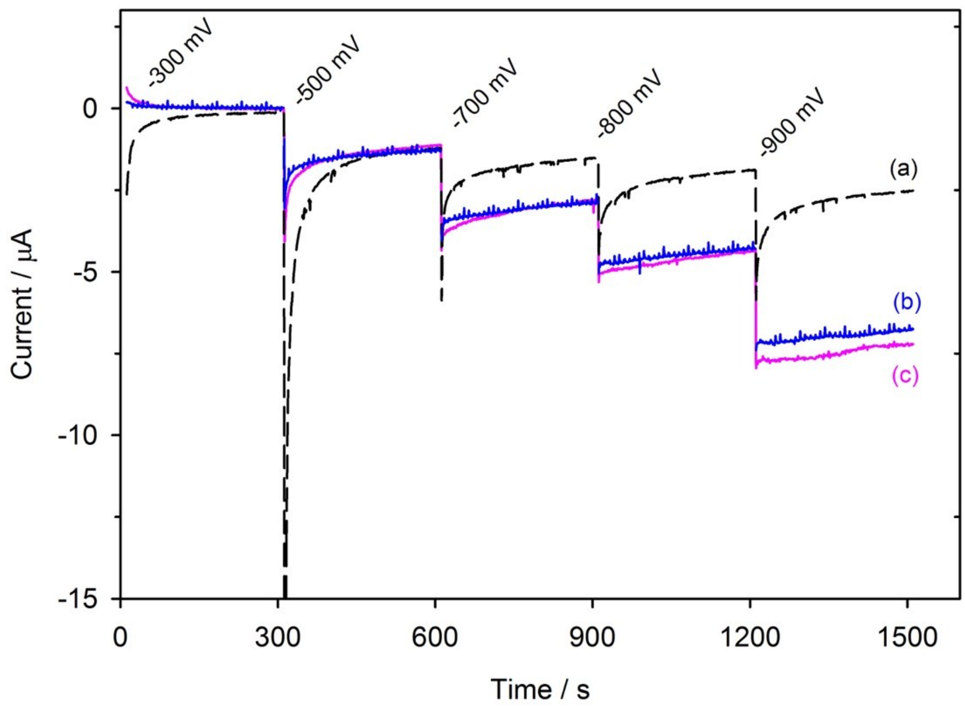


**Figure S5. Current-time trace for a control experiment with Eu-L in the absence of protein under an Ar atmosphere (a); and wild type MoFe protein (2.0 nmol) in the presence of Eu-L in solution saturated with $N_2$ (b) and Ar (c). A sequence of potential steps was applied at the times indicated. The difference in current between traces (b) and (c) and trace (a) reflects the electrocatalytic contribution from the wild type MoFe protein.**

**Evidence for spectral features below 1900 cm$^{-1}$ for the β-99$^{Phe\rightarrow His}$ variant at low potentials**

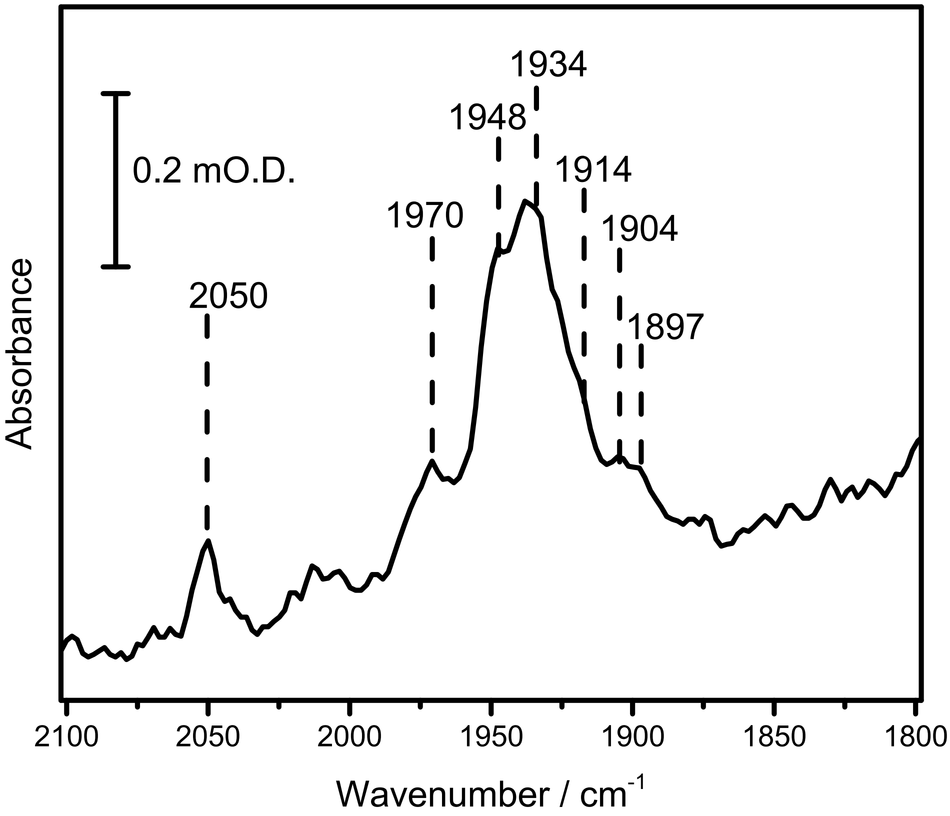


**Figure S6. Difference spectrum showing an increase in concentration of all CO bands at more negative potentials for the β-99$^{Phe\rightarrow His}$ variant. Spectra are processed as -1000 mV minus -800 mV, calculated from the data shown in Figure 5(d) before baseline correction. Lower wavenumber features below 1900 cm$^{-1}$ become evident in this difference spectrum.**

**Potential-dependent reversibility of CO binding to wild type MoFe protein**

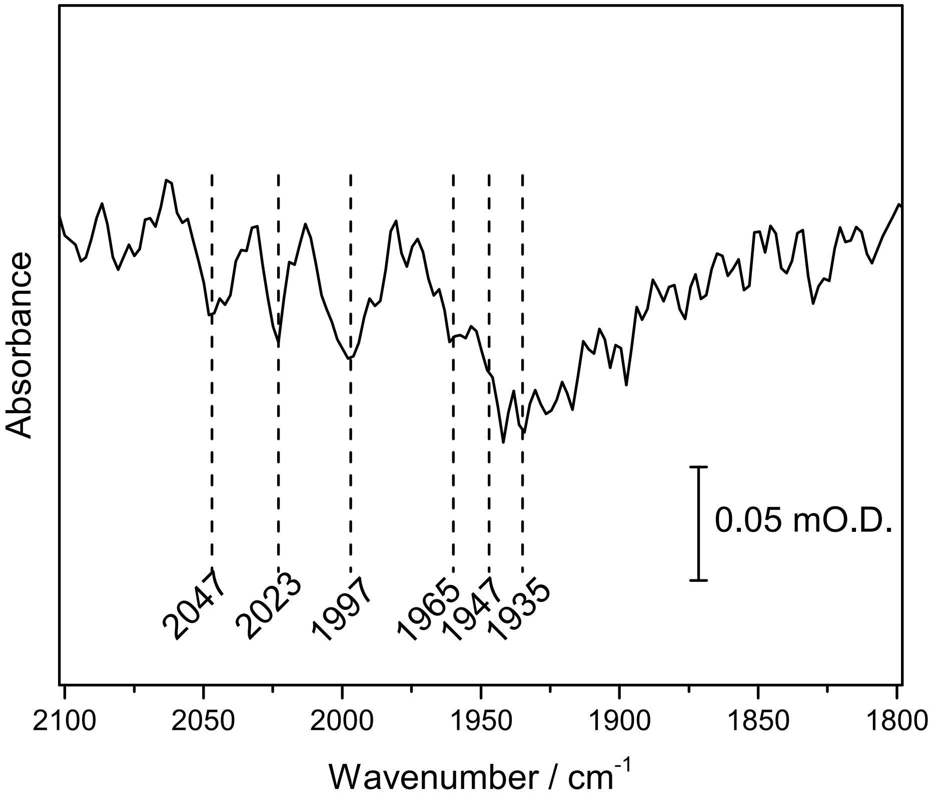


**Figure S7. Potential-dependent reversibility of CO binding to wild type MoFe protein. A difference spectrum recorded after the experiment shown in Figure 5b of the main text, following flushing out CO with $N_2$ and stepping the potential to -100 mV. The difference spectrum is presented as -100 mV under $N_2$ *minus* -1000 mV under CO. Negative bands therefore represent species that have been depleted following re-oxidation of the MoFe protein under $N_2$.**

**Supplementary References**

(1) B. K. Burgess, D. B. Jacobs, E. I. Stiefel, *Biochim Biophys Acta,* 1980, 614, 196.

(2) J. Christiansen, P. J. Goodwin, W. N. Lanzilotta, L. C. Seefeldt, D. R. Dean, *Biochemistry,* 1998, 37, 12611.

(3) J. Wiig, C.-C. Lee, A. Fay, Y. Hu, M. W. Ribbe, In Nitrogen Fixation, M. W. Ribbe Ed., Humana Press, 2011, 766, 93.

(4) A. J. Healy, P. A. Ash, O. Lenz and K. A. Vincent, *Phys. Chem. Chem. Phys.*, 2013, **15**, 7055.

(5) D. Lukoyanov, N. Khadka, Z.-Y. Yang, D. R. Dean, L. C. Seefeldt, B. M. Hoffman, *J. Am. Chem. Soc.*, 2016, **138**, 1320.
